# Effects of a Multi-Strain Lactic and Propionic Acid Bacteria Inoculant on Silage Quality, Methane Emissions, Milk Composition, and Rumen Microbiome

**DOI:** 10.3390/ani15182740

**Published:** 2025-09-19

**Authors:** Segun Olorunlowu, Pola Sidoruk, Julia Sznajder, Jakub Szczesny, Dorota Lechniak, Piotr Pawlak, Marcin Ryczek, Haihao Huang, Lingyan Li, Agung Irawan, Jolanta Komisarek, Malgorzata Szumacher-Strabel, Adam Cieslak

**Affiliations:** 1 Department of Animal Nutrition, Poznań University of Life Sciences, Wołyńska 33, 60-637 Poznań, Poland; olorunlowusegunabraham@uppoz.onmicrosoft.com (S.O.); pola.sidoruk@up.poznan.pl (P.S.); jula.sznajder@gmail.com (J.S.); jakub.szczesny422@gmail.com (J.S.); malgorzata.szumacher@up.poznan.pl (M.S.-S.); 2 Department of Genetics and Animal Breeding, Poznań University of Life Sciences, Wołyńska 33, 60-637 Poznań, Poland; dorota.cieslak@up.poznan.pl (D.L.); piotr.pawlak@up.poznan.pl (P.P.); marcin.ryczek@up.poznan.pl (M.R.); 3 College of Animal Science and Veterinary Medicine, Heilongjiang Bayi Agricultural University, Daqing 163000, China; haihao.huang@gmail.com (H.H.); llytiger@163.com (L.L.); 4 Vocational School, Universitas Sebelas Maret, Surakarta 57126, Indonesia; a.irawan@staff.uns.ac.id; 5 Department of Animal Breeding and Product Quality Assessment, Poznań University of Life Sciences, Złotniki, Słoneczna 1, 62-002 Suchy Las, Poland; jolanta.komisarek1@up.poznan.pl

**Keywords:** silage, fermentation quality, rumen fermentation, lactation performance, methane emission, fatty acid

## Abstract

Ensiling grass with microbial inoculants is a promising strategy to enhance forage quality, animal performance, and environmental sustainability. This study evaluated the effects of a multi-strain inoculant (*Lactobacillus plantarum*, *L. buchneri*, *Propionibacterium acidipropionici*, and *P. thoeni*) on silage fermentation, nutrient digestibility, milk production, methane emissions, and rumen microbiota in dairy cows. In a 2 × 2 crossover design, 24 lactating Polish Holstein–Friesians were fed total mixed rations differing only in grass silage treated with or without inoculant. Inoculated silage had lower pH (4.56 vs. 5.06; *p* = 0.02) and higher crude protein (129 vs. 111 g/kgDM; *p* < 0.05). Cows fed inoculated silage showed higher ruminal propionate (28.3 vs. 26.3 mM; *p* = 0.03), reduced ammonia (7.61 vs. 8.67 mM; *p* = 0.02), and fewer protozoa (1.21 vs. 1.66 × 10^5^/mL; *p* = 0.03). Nutrient digestibility improved (*p* < 0.05), while methane emissions declined both per cow (368 vs. 397 g/d; *p* = 0.01) and per kgDMI (15.1 vs. 16.5; *p* = 0.01). Milk yield increased (*p* = 0.04), and the fatty acid profile improved. Our study revealed that cows fed inoculated silage had higher nutrient digestibility, lower methane emissions, and microbial shifts in the rumen detected by 16S rRNA sequencing (*p* < 0.05).

## Simple Summary

Dairy farming must balance milk production with environmental sustainability, as cows emit methane, and silage quality affects feed efficiency. In this study, we tested whether a mix of beneficial bacteria to grass during ensiling (the storage process) could improve feed quality, cow performance, and reduce methane. We found out that cows fed the inoculated silage produced more milk with healthier fats, digested feed more efficiently, and emitted less methane. Using DNA-based methods, we also discovered subtle changes in the microbial community inside the cow’s stomach that were linked to these improvements. These findings suggest microbial inoculants offer a natural way to enhance dairy productivity while reducing environmental impact.

## 1. Introduction

With rising global demand for dairy products, sustainable dairy production has become essential [1,2]. However, challenges like feed cost [3], weather variability, forage degradation [4], and poor fodder preservation [5] highlight the need for high-quality conserved forages like silage to ensure year-round productivity and sustainable dairy production [6,7]. Improving silage quality is vital, as it directly influences feed efficiency, milk yield, and environmental impact [8]. Moreover, better silage preservation not only reduces feed losses and boosts nutrient use but also supports animal health and farm profitability [9,10]. These improvements are closely linked with enhancing forage digestibility, which is one of the key strategies to reduce emissions per unit of milk, given that enteric methane (CH_4_) contributes about 6.3% of anthropogenic greenhouse gases [11].

Grass silage fermentation is an anaerobic process primarily driven by lactic acid bacteria (LAB), which convert water-soluble carbohydrates into lactic acid, lowering pH and inhibiting spoilage microbes [11,12]. This preservation process retains the silage’s nutritional value and palatability by preventing the growth of undesirable microbes, such as clostridia [13]. *Lactobacillus plantarum* is a common homofermentative LAB used as a silage inoculant to accelerate lactic acid production and achieve a rapid, stable fermentation [14,15]. By dominating the epiphytic (naturally occurring) flora, such inoculants ensure a more controlled and efficient fermentation, thereby reducing losses in dry matter and forage quality during ensiling [16,17,18].

While homofermentative LAB such as *L. plantarum* efficiently produce lactic acid and preserve fermentable energy, silage treated with LAB is more prone to aerobic spoilage at feed-out, as lactic acid offers limited inhibition of spoilage organisms [18]. Under aerobic conditions, yeasts and molds metabolize lactic acid, leading to heating and nutrient loss [19]. To improve aerobic stability, heterofermentative LAB such as *L. buchneri* are used to convert some lactic acid into acetic acid, which, though less efficient at preserving sugars, strongly inhibits spoilage microbes [20,21]. Propionic acid bacteria (e.g., *Propionibacterium acidipropionici* and *P. thoeni*) further enhance stability by producing propionic acid, known for its antifungal properties [22,23]. Adding *Propionibacterium*, alone or with LAB, improves aerobic stability by suppressing yeasts and molds [18]. A multi-strain inoculant combining *L. plantarum*, *L. buchneri*, *P. acidipropionici*, and *P. thoeni* leverages their complementary roles to improve fermentation and resist spoilage.

In addition, improving silage with multi-strain inoculants can also reduce greenhouse gas emissions linked to both the ensiling process and methane output per unit of feed intake [24]. LAB such as *L. plantarum* increases lactic acid in silage, which rumen microbes convert to propionate, a pathway that uses hydrogen and reduces methane formation [25]. Levenson et al. [26] and Vyas et al. [27] also reported that propionic acid bacteria influence methanogenesis by shifting rumen fermentation toward propionate production, which reduces the availability of hydrogen (H_2_) for methanogens, thereby lowering CH_4_ emissions. High-quality silage also improves palatability and nutrient content, boosting dry matter intake and milk yield or weight gain in cows [28]. Additionally, LAB-inoculated silage enhances milk’s fatty acid profile, increasing polyunsaturated and reducing saturated fatty acids compared to control-fed animals [6].

Although microbial inoculants are gaining interest, most studies assess either fermentation or animal performance alone, with limited integration of environmental indicators such as CH_4_ emissions. Research on multi-strain inoculants, combining homofermentative LAB, heterofermentative LAB, and propionic acid bacteria, is still limited, especially regarding their combined effects on silage quality, rumen function, methane mitigation, milk production and composition, and 16S rRNA gene sequencing of the microbiota in rumen fluid. This study addresses that gap by evaluating a combined inoculant (*L. plantarum*, *L. buchneri*, *P. acidipropionici*, *P. thoeni*) in dairy cows. This study is among the first to assess the impact of these strains of lactic and propionic acid bacteria inoculant on improvements in silage quality, rumen parameters, total tract digestibility, methane mitigation, rumen microbiota, milk yield, and fatty acid profile, underscoring the novelty of a multi-trait evaluation. We hypothesized that this formulation would enhance silage quality, improve fermentation and digestibility, reduce methane emissions, and yield more milk with a healthier fatty acid profile.

## 2. Materials and Methods

This research was carried out within the Climate Care Cattle Farming Systems (CCCFarming) project, as part of the international grant 2018 JOINT CALL FACCE ERA-GAS, SusAn, ICT-AGRI2) and is part of Gorzowska Catch Crop Grass Mixture analysis, including feeding value and reducing the negative impact of animal production on the environment. All experimental procedures were performed according to the National Ethical Commission for Animal Research guidelines (Ministry of Science and Higher Education, Poland). This study was approved by the Local Ethics Commission (decision no. 44/2023). The number of ruminally cannulated cows used in this study was restricted by the terms for ethical approval. Consent was obtained from the farm owner before the trial, and the purpose and potential consequences of the research were fully explained.

### 2.1. Experimental Design and Diet Preparation

This experiment was conducted on a private dairy cow farm in the village of Turkowo, Nowy Tomyśl County, Greater Poland Voivodeship, Poland. Twenty-four lactating Polish Holstein-Fresian dairy cows (633 ± 42 kg body weight, 144 ± 19 d in milk, 33.6 ± 1.8 kg/d milk production, and 2.1 ± 0.6 lactation number) were used for this study, four of which were cannulated, as permitted for ethical approval. Based on diet composition, the cows were randomly allotted to two experimental groups: control and experimental. Both diets were formulated to contain the same ingredients and chemical composition, differing only in the grass silage. Each experimental group had twelve cows (ten with no canula and two cannulated). Cows with no canula were housed in a free stall, while the canulated animals were housed in individual tie stalls with rubber mats and a controlled feeding system. Both groups were fed the diets for 34 days; thereafter, they were switched to the other treatment for another 34 days, forming a 2 × 2 crossover design. The first 26 days of each experimental period were for diet adaptation, and the remaining 8 days were for data collection. Diets were formulated according to INRA et al. [29] standards to meet the nutrient requirements of lactating dairy cows. The SmartFeed software (SmartFeed, Puławy, Poland) was used as a formulation tool to balance the rations. The ration was served twice a day (at 6:00 h and 18:00 h) as a total mixed ration (TMR) on the feeding table assigned to each group. Both TMRs contained (g/kg of DM): maize silage (320); grass silage (230; with or without inoculant); beet pulp (73), commercial concentrate (358; POWER 25, 25% CP, DeHeus, Ede, The Netherlands); fodder chalk (8; Holcim fodder chalk, 94% CaCO_3_, 37.6% Ca); mineral and vitamin premix (12; Bestermine Biocomplex, DeHeus, Ede, The Netherlands). Cows had ad libitum access to clean water.

This study used Gorzowska Catch Crop Grass Mixture (50% Italian ryegrass, 30% Incarnate clover, and 20% Winter vetch) as the grass silage. The silage was cultivated on 40 ha of arable land and was harvested at an early heading. After harvesting, the silage was wilted for 2 days (~42% DM), chopped into 3–4 cm pieces with Claas Jaguar 870 forage harvester, and divided into two large silos (75 tons each), with one prepared without inoculant (control) and the other treated with the multi-strain inoculant (experimental). Each silo represented a single replicate for its respective treatment. The experimental silo was treated with the combined inoculant using the DioDan commercial product (DDCI JHJ, Nowa Wieś, Poland). The inoculant strains are indexed in the Polish Collection of Microorganisms (PCM) database, a public biological resource recognized under the Budapest Treaty for patent purposes, allowing depositors to use the PCM for safeguarding their microbial inventions. The bacterial strains in the inoculant are *L. buchnerii* (JHJ006-PCM: B/00280), *L. plantarum* (JHJ003-PCM: B/00166), *P. acidipropionici* (JHJP02-PCM: B/00285), and *P. thoenii* (JHJP01-PCM: B/00284), with a total bacterial count of 3 × 10^9^ CFU/g, each strain comprising 25% of the total count. The carriers within the inoculant were primarily glucose/maltodextrin. As recommended by the manufacturer, 500 g of the inoculant was mixed with 50 L of non-chlorinated water to prepare the aqueous solution. One liter of the prepared solution (bacteria count—3 × 10^10^ CFU per liter) was then used to prepare 1 ton of ensiled material. The 2 silage groups were then compacted. After compaction, each pile was immediately covered with a single layer of black-and-white silage film (Silofolien), edged with soil, overlaid with a protective net, weighed down with tires, and incubated at ambient temperature (29–36 °C) for 90 days. After the incubation period, grass silage samples were collected.

### 2.2. Data Collection

Samples of the silages were collected on the opening day and stored for the analysis of the chemical composition and fatty acid profile. Silage samples were taken from different locations of each silo at random. Thereafter, these samples were mixed for homogeneity. From these homogeneous mixtures, three subsamples for each treatment were taken to analyze for chemical and fermentation analyses, and each analysis was performed in duplicate. The dry matter intake was measured by weighing the offered feed and leftovers from the feeding table of each group.

For the determination of total-tract nutrient degradability coefficients, feed intake, feed refusals, and fecal output were recorded daily for individual ruminally cannulated dairy cows housed in tie-stalls during both periods when they were kept in individual tie stalls and were placed in respiration chambers. Dry matter intake (DMI) was also monitored while the cows were housed in the respiration chambers and was included in the daily calculations. Subsamples of feed and feces (approximately 5% wt/wt) were collected daily and stored at −20 °C until analysis of dry matter (DM), organic matter (OM), and crude protein (CP). Total-tract nutrient digestibility was calculated using the following equation:

$$Digestibility\;(g/kg\;DM)=\frac{nutrient\;intake-nutrient\;left\;in\;feces}{nutrient\;intake}$$

Milk samples were collected twice daily at 5:30 and 17:30 h on days 1, 3, 6, and 8 of each experimental phase in a herringbone milking parlor with eight milking units. Each treatment group consisted of 12 cows (*n* = 12 biological replicates). For each cow, morning and evening samples were pooled proportionally according to milk yield to obtain one representative daily sample per cow. These daily samples were analyzed individually, and compositional analyses were performed in duplicate (technical replicates). These samples were used to assess lactation parameters. Milk production was measured using a milk meter (WB Ezi-Test Meter 33 kg; Tru-Test Limited, Manukau, New Zealand). Finally, a 15 mL sample was prepared for further analysis.

Rumen fluid was collected from the four cannulated cows (*n* = 2 biological replicates per treatment) for four days (1, 3, 6, and 8), when the cows were not in the respiration chambers. The collection was performed before feeding the TMR at 6:00 h, then 3 and 6 h after feeding. Approximately 500 mL of rumen content was collected from each cow at every collection time from three locations (top, bottom, and middle) of the midventral sac. Thereafter, the contents were filtered through a two-layered cheesecloth. The samples were analyzed for pH, VFA concentrations, and protozoa counts (in duplicate). 6 mL of the RF was used for FA analysis; 3.6 mL of the RF sample was treated with 0.5 mL of 10% formic acid for the volatile fatty acid (VFA) analysis; 1 mL of RF was mixed with 6 mL of 4% formalin for the protozoa count. All the samples were prepared in two repeats and stored at 4 °C in the laboratory for further analysis, except for protozoa count, which was carried out immediately after sampling at room temperature to minimize the risk of changes associated with prolonged storage.

Methane (CH_4_) concentration was measured using cannulated dairy cows by two mobile respiratory chambers with two separate nondispersive infrared spectroscopy (NDIR) systems (one per chamber) operating in the near-infrared spectrum (detector 1210 Gfx Servomex 4100, Servomex, Crowborough, UK). Air samples were drawn continuously at a rate of 0.6 L/min, dried using a TC-standard gas cooler (Buhler Technologies, Ratingen, Germany), and analyzed in cuvettes with optical track lengths of 540 mm for CH_4_. The system was calibrated before measurements using nitrogen 5.0 (99.999% purity) and a reference gas containing 1210 ppm CH_4_ in nitrogen. Concentrations of CH_4_ were recorded every 2 s. CH_4_ emission (g/d) was calculated automatically by the system software based on the difference between outlet and inlet concentrations (ppm), corrected for airflow (m^3^/h), and converted into mass flow using methane’s density constant (0.0006797 g/m^3^ per ppm under standard temperature and pressure (0 °C, 1013 hPa)), as described by Sypniewski et al. [30] with additional corrections applied to the actual temperature and pressure recorded during the experiment. For the measurement, cows were placed in the respiration chambers immediately after the morning and evening milking and remained there for three hours. During chamber measurements, cows had continuous access to feed and water. After each measurement period, they were returned to their individual tie-stalls. Finally, measurements were taken for each cow for 6 h at 2 s measuring intervals. During the 8-day data collection period, each cow underwent two measurement sessions. The results were then multiplied by a factor of 4 to obtain the daily methane emissions.

### 2.3. Chemical Analysis

Silage, diet, and fecal samples were analyzed following the procedure of AOAC [31] for dry matter (DM; method no. 934.01), crude protein (CP; Kjel-Foss Automatic 16,210 analyzer, Foss, Hillerød, Denmark; method no. 976.05), crude fat (CF; Soxhlet System HT analyzer; Foss, Hillerød, Denmark; method no. 973.18). The neutral detergent fiber (NDF) with amylase and sodium sulfite, expressed without residual ash, was analyzed using the Fibretec 1020 Analyzer (Foss, Analytical AB, Höganäs, Sweden) following the procedure of Van Soest et al. [32]. Organic matter (OM) was calculated by subtracting ash from DM.

The concentration of organic acids in grass silages was determined using high-performance liquid chromatography (HPLC; Waters-Acquity Arc, Waters Alliance, MA, USA) equipped with a Waters 2489 UV/Vis detector set to 210 nm. The analytical procedure followed the method described by Kostulak-Zielińska and Potkański [33]. Separation was performed on an Aminex HPX-87 column (Bio-Rad Laboratories, Hercules, CA, USA) using 0.005 mol/L sulfuric acid as the mobile phase. The column temperature was maintained at 35 °C.

The pH of silage was measured immediately after silo opening using a pH meter (Elmetron, Type CP-104, Zabrze, Poland). Rumen fluid pH was determined at the time of sampling (before feeding and at 3 and 6 h post-feeding) using the same pH meter. The ammonia concentration in RF was determined using the colorimetric Nessler method as prescribed by Szczechowiak et al. [34]. Protozoa counts in RF were performed under a light microscope (Primo Star 5, Zeiss, Jena, Germany) using an appropriate volume (10 μL for *Ophryoscolecidae* and 100 μL for *Isotrichidae*) of the formalin-fixed RF. The mixture was well mixed and then counted under a light microscope (×10 objective) by tracking back and forth across the area of the liquid as described by Coleman [35] after the protozoa identification as described by Dehority [36]. VFA in RF was measured following the method described by [34] with minor improvements. RF samples were centrifuged at 16,800 rcf for 5 min at 4 °C (Mikro 200R Refrigerated Centrifuge; Hettich, Tuttlingen, Germany) and transferred to a 1.5 mL sample vial. Sample vials were placed in the autosampler (CP 8400; Varian, Sugarland, TX, USA) and analyzed using gas chromatograph (GC Varian CP 3380, Sugarland, TX, USA) equipped with a packed column (10% Chromosorb Wax 20M-TPA, mesh size 100/120, 2 m × 1/8 in, Supelco, Bellefonte, PA, USA), and a flame ionization detector (FID). The amount of 1 µL of sample in splitless mode was injected into the column. The analysis lasted for 30 min with a 7 mL/min hydrogen carrier gas flow rate, using a 123 °C constant oven temperature, 200 °C for the injector, and 250 °C for the detector. The identification of VFAs peaks, both qualitatively and quantitatively, was carried out using external standards created by combining pure, individual VFAs sourced from Fluka (Sigma Aldrich, St. Louis, MO, USA). The data obtained were processed with MS Work Station 5.0. software (Varian, Sugarland, TX, USA). The content of individual VFAs was expressed in mM values.

Composite milk samples were analyzed for basic milk constituents using an infrared analyzer (Milko-Scan 255 A/S N; norm PN-ISO 9622:2015-09; Foss, Hillerød, Denmark). The urea concentration in milk was determined in an accredited milk quality laboratory using infrared spectrometry and a CombiFoss 6000 analyzer (Foss, Hillerød, Denmark).

Samples for Fatty acid (FA) analysis were collected and stored at −20 °C immediately after sampling. All FA analyses were performed a month after collection to avoid degradation or compositional changes during storage. FA profile of silage and milk was determined following the method described by Szczechowiak et al. [34] with minor improvements. Hydrolysis was performed by adding 3 mL of 2 M NaOH to 0.1 g of silage and 0.25 g of milk, followed by incubation for 40 min at 90 °C. Extraction was conducted by adjusting the pH to 2 using 4 M HCl solution and repeating the following steps three times: adding 3 mL of diethyl ether, mixing for 30 min (Intelli-Mixer RM-2L; ELMI, Riga, Latvia) and centrifuging at 4200 rcf for 1 min in 20 °C (Eppendorf 5804R; Eppendorf, Hamburg, Germany) to obtain the organic phase, which was moved to the sample concentrator in 35 °C under the nitrogen atmosphere (Techne DB-3 Sample Concentrator; Techne Inc., Burlington, NJ, USA). The derivatization process was completed by adding the molecular sieves (4A type; Chempur, Poland) to tubes with extracted fat, then boiling for 3 min in 2 mL of 0.5 M NaOH in methanol and adding 3 mL of 10% boron trifluoride-methanol solution (Supelco, Bellefonte, PA, USA), continued boiling for 4 min and cooling to room temperature. The process was finished by adding 7 mL of 0.34 M NaCl and 0.5 mL of n-hexane, mixing for 45 min, and centrifuging at 4200 rcf for 1 min at 20 °C to obtain the organic phase, which was moved to a 1.5 mL sample vial with a 400 µL micro-insert. Sample vials were placed in the autosampler (CP-8400; Bruker Scion, Billerica, MA, USA) and analyzed using gas chromatograph (456-GC; Bruker Scion, Billerica, MA, USA) equipped with capillary column (100 m × 0.25 mm; overlaid with 0.25 µm Agilent HP; Chrompack CP7420; Agilent Technologies, Santa Clara, CA, USA) and flame ionization detector (FID). The amount of 1 µL of sample with an initial 10 split ratio and a 20 split ratio in 1.75 min of analysis was injected into the column. The analysis lasted for 55 min with a 1.3 mL/min flow rate of hydrogen carrier gas, using the following oven temperature program: initially 120 °C for 7 min, then increasing at 7 °C /min to 140 °C, holding for 10 min, and then increasing at 4 °C /min to 240 °C. Injector and detector temperatures were set to 250 °C. Fatty acids were identified by comparing the retention times with an external FAME standard (37 FAME Mix, Sigma Aldrich, Bellefonte, PA, USA) and conjugated isomers of linoleic acid standard (CLA methyl ester, Sigma Aldrich, Bellefonte, PA, USA) using the CompassCDS software Version 3.0.1(Bruker Scion, Billerica, MA, USA). The content of individual fatty acids was expressed as FAME in g/100 g FAME values.

### 2.4. Microbial DNA Extraction, Sequencing, and Bioinformatics Analysis

Microbial DNA was extracted from pooled rumen fluid samples, and each extracted sample was subjected to one sequencing run. 0.5 mL of rumen fluid was transferred to LoBind 1.5 mL Eppendorf tubes and gently centrifuged (500× *g* for 1 min.). Next, the supernatant was centrifuged at 14,000× *g* for 10 min to pellet the microorganisms. PureLink Microbiome DNA Purification Kit (ThermoScientific, Waltham, MA, USA; A29790) was used for pure DNA isolation. Supernatant was discarded, and the pellet was resuspended in 800 µL of S1, Lysis Buffer following transfer to the Bead Tube. Next steps were processed according to the manufacturer’s protocol. Firstly, 100 µL of S2, Lysis Enhancer was added, vortexed briefly, and incubated at 65 °C for 10 min. Samples were homogenized by bead beating for 10 min at maximum speed using a vortex and centrifuged at 14,000× *g* for 2 min. The 400 µL of supernatant was transferred to a clean microcentrifuge tube, and 250 µL of S3, Cleanup Buffer was added and vortexed immediately. After centrifugation, 500 µL of the supernatant was transferred to 900 µL of S4, Binding Buffer in a new clean Eppendorf tube. 700 µL of the sample was placed on a spin column-tube and centrifuged at 14,000× *g* for 1 min twice. Next steps involved S5-Wash buffer sample cleanup and removal of wash buffer. Finally, 100 µL of S6, Elution Buffer was added to the column and incubated at room temperature for 1 min. The purified DNA was obtained by final centrifugation of the spin column-tube at 14,000× *g* for 1 min. The purity and quality of DNA were determined using a Nanodrop One Spectrophotometer (ThermoScientific, Waltham, MA, USA).

Libraries were constructed for the hypervariable V3-V4 region for the 16S rRNA gene using the manufacturer’s protocol (Illumina, San Diego, CA, USA) for the Illumina MiSeq System–MiSeq Reagent Kit v3 (600 cycles). The gene-specific sequences are selected from Klindworth et al. [37] and included in Illumina guidelines for 16S Metagenomic Sequencing Library Preparation. Amplicons were prepared using Pyromark PCR Kit (Qiagen, Venlo, The Netherlands) following the AMPure XP bead purification protocol (Beckman Coulter, Brea, CA, USA). Samples were indexed using Nextera XT Index Kit v2 set A and measured using Qubit ds DNA HS Assay Kit (Invitrogen by ThermoScientific, Waltham, MA, USA) on Qubit fluometer 2.0. Libraries were pooled to reach a final concentration of 6 pM and 5% PhiX control and sequenced via the MiSeq Illumina platform at the Department of Genetics and Animal Breeding.

### 2.5. Bioinformatics and Microbiota Community Profiling

Microbiome composition was analyzed based on 16S rRNA gene sequencing data processed using the DADA2 pipeline (version 1.34.0) in R (version 4.4.2) for quality filtering, denoising, chimera removal, and generation of amplicon sequence variants (ASVs). Sequencing quality was high, with a global median Q-score of 35 across all samples. Sequencing generated an average of ~161,000 raw reads per sample. After quality filtering, approximately 143,000 reads per sample were retained, and ~24,000 non-chimeric sequences were obtained for downstream analysis. Taxonomic classification was performed to determine relative abundances at both phylum and genus levels. In total, 11,244 amplicon sequence variants (ASVs) were identified. The majority of ASVs could be assigned to known phyla; only 163 ASVs (1.45%) remained unassigned at the phylum level. To normalize the data, the ASV table was rarefied to the minimum sequencing depth across samples using the *rarefy_even_depth* function from the *phyloseq* package (version 1.50.0). Rarefied data were used for downstream diversity analyses (alpha and beta diversity. Alpha diversity was assessed with the Simpson index to evaluate within-sample diversity and dominance. Statistical differences in the Simpson index were assessed by the Wilcoxon rank-sum test. Beta diversity was calculated using Jaccard and Bray–Curtis distance metrics to compare microbiome structures between the control and experimental groups. Statistical differences in community composition were tested using permutational multivariate analysis of variance (PERMANOVA) implemented via the Adonis function in the *vegan* R package (version 2.6-10), with 999 permutations. Statistical significance was set at *p* < 0.05.

### 2.6. Statistical Analysis

Before analysis, all data were tested for normality using the Shapiro–Wilk test. All data followed a normal distribution except for the rumen parameters and methane emissions. Rumen parameters and methane emission data were analyzed using independent Wilcoxon rank-sum tests in R statistical software (version 4.4.2), with group means compared using the PROC TTEST procedure. Silage chemical composition, milk yield, milk composition, and fatty acid profile of milk were analyzed using the General Linear Model (GLM) procedure in SAS software (version 9.1, SAS Institute Inc., Cary, NC, USA), with treatment (control vs. inoculated silage) and period included as fixed effects and cow as a random effect to account for the 2 × 2 crossover design; when significant treatment effects were detected, means were separated using Duncan’s multiple range test.

## 3. Results

Table 1 shows the effect of the combined inoculant on the fermentation characteristics, chemical, and fatty composition of grass silage. The inoculant significantly lowered silage pH (4.56 vs. 5.06; *p* = 0.02) and increased crude protein content (129 vs. 111 g/kg DM; *p* < 0.05), lactic acid (56.8 vs. 78.3; *p* = 0.03), and propionic acid (4.61 vs. 7.62; *p* = 0.01) when compared with the control. While there was no significant effect on other chemical constituents (*p* > 0.05), the DM, OM, and NDF were reduced. Likewise, there was no significant impact on the fatty acid profile of the silage (all *p* > 0.05).

The chemical composition and fatty acid (FA) profile of the total mixed rations (TMRs) fed to the control and experimental groups are presented in Table 2. After incorporating 230 g/kg of grass silage into the TMRs, no significant differences were observed in DM, OM, CP, and NDF content between the two diets (*p* > 0.05), although the experimental TMR showed a numerically higher CP (164 vs. 156 g/kg DM) than the control.

Table 3 shows the effect of the combined inoculant on the basic rumen parameters. The rumen fluid from inoculant-treated silage showed higher pH (6.06 vs. 5.83; *p* = 0.03) and propionate concentration (28.3 vs. 26.3 mM; *p* = 0.03), coupled with a reduced A:P ratio (2.60 vs. 2.87; *p* < 0.01). Isovalerate (1.57 vs. 2.02 mM; *p* = 0.02), valerate (1.41 vs. 1.82 mM; *p* < 0.01), and ruminal NH_3_ (7.61 vs. 8.67 mM; *p* = 0.02) were lower in the experimental group, while total VFA and butyrate remained unchanged (*p* > 0.05). Protozoal counts shifted, with *Isotrichidae* increasing and *Ophryoscolecidae* decreasing, yielding an overall reduction in total protozoa (121 vs. 166 × 10^3^/mL; *p* = 0.03).

Table 4 reveals the influence of the inoculant on the lactation performance of dairy cow. The dry matter intake was similar across treatments (24.0 vs. 24.5 kg/d; *p* = 0.19), but the inoculant increased both milk yield (35.3 vs. 33.5 kg/d; *p* = 0.04) and energy-corrected milk (37.2 vs. 35.3 kg/d; *p* = 0.05). Among basal milk components, the CP (3.87 vs. 3.64%; *p* = 0.01) and lactose (4.76 vs. 4.64%; *p* = 0.01) increased, while fat and DM remained unchanged (*p* > 0.05). The milk urea concentration was also higher with inoculant (223 vs. 203 mg/L; *p* = 0.04). CP (1.36 vs. 1.22 kg; *p* = 0.01), casein (1.07 vs. 0.98 kg; *p* = 0.01), and lactose (1.68 vs. 1.56 kg; *p* = 0.01) in the daily milk solids were similarly elevated.

Table 5 shows the effect of the combined inoculant on methane production and total tract digestibility. Digestibility improved with inoculant: DM (650 vs. 629 g/kg DM; *p* = 0.04), OM (712 vs. 690 g/kg DM; *p* = 0.04), and CP (636 vs. 596 g/kg DM; *p* = 0.01) were all higher than the control group. Concurrently, methane emissions declined both per cow (368 vs. 397 g/d; *p* = 0.01) and per unit intake (15.1 vs. 16.5 g/kg DMI; *p* = 0.01).

Table 6 shows the effect of the combined inoculant on the fatty acid profile of milk. Among SFA, C14:0 (14.6 vs. 14.1%; *p* = 0.02) and C18:0 (10.1 vs. 9.37%; *p* = 0.03) increased while C16:0 (32.9 vs. 36.7%; *p* < 0.01) reduced. MUFA changes included elevated trans-10 (0.56 vs. 0.51%; *p* = 0.02) and trans-11 isomers (1.03 vs. 0.79%; *p* < 0.01). Amidst the PUFA, C18:2 cis-9, trans-11 (0.60 vs. 0.48%; *p* < 0.01) and C18:2 trans-10, cis-12 (0.15 vs. 0.14%; *p* < 0.01) increased, while C 18:2 cis-9, 12 (2.60 vs. 2.68; *p* = 0.02) and C18:3n-3 (0.40 vs. 0.49%; *p* < 0.01) decreased. Overall, total SFA declined (65.2 vs. 67.2%; *p* = 0.02) while UFA increased (34.8 vs. 32.8%; *p* = 0.02), MUFA rose (30.3 vs. 28.5%; *p* < 0.01), and LCFA expanded (42.4 vs. 39.5%; *p* < 0.01). The n-6/n-3 ratio also increased (10.1 vs. 8.97; *p* < 0.01), and desaturation index C14:1 reduced (0.09 vs. 0.11; *p* = 0.01).

**Figure 1:**
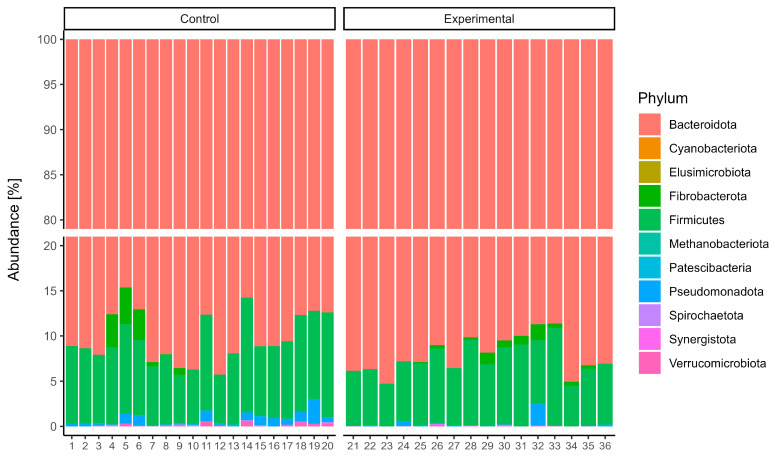
Relative Abundance of Microbial Taxa at the Phylum Level of cows fed a ration containing grass with and without combined inoculant.

**Figure 2:**
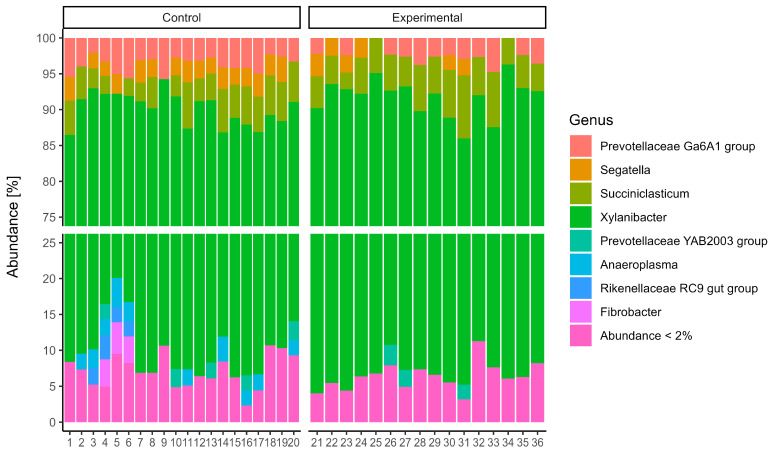
Relative Abundance of Microbial Taxa at the Genus Level of cows fed a ration containing grass with and without combined inoculant.

**Figure 3:**
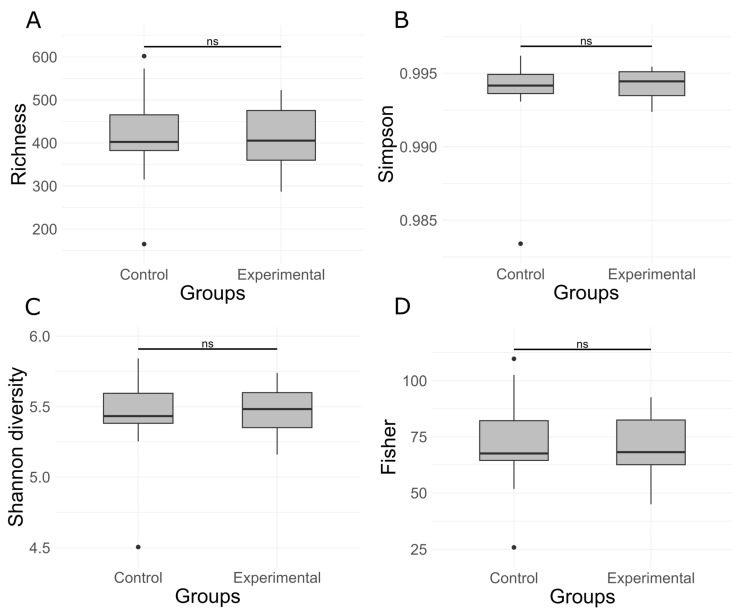
Alpha Diversity of Microbial Communities Based on (**A**) Observed Richness, (**B**) Simpson Diversity Index, (**C**) Shannon Diversity Index, and (**D**) Fisher Index. (ns—not significant, *p* > 0.05).

**Figure 4:**
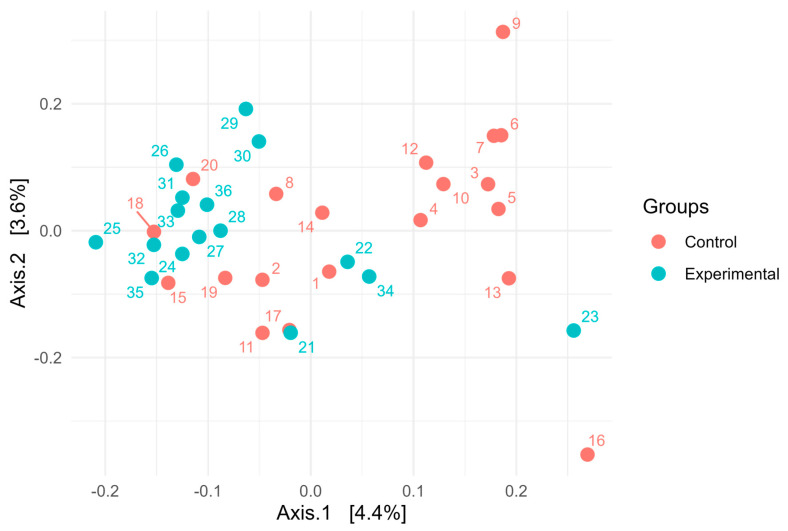
Principal Coordinates Analysis (PCoA) of Microbial Communities Based on Jaccard Distance of cows fed a ration containing grass with and without combined inoculant.

**Figure 5:**
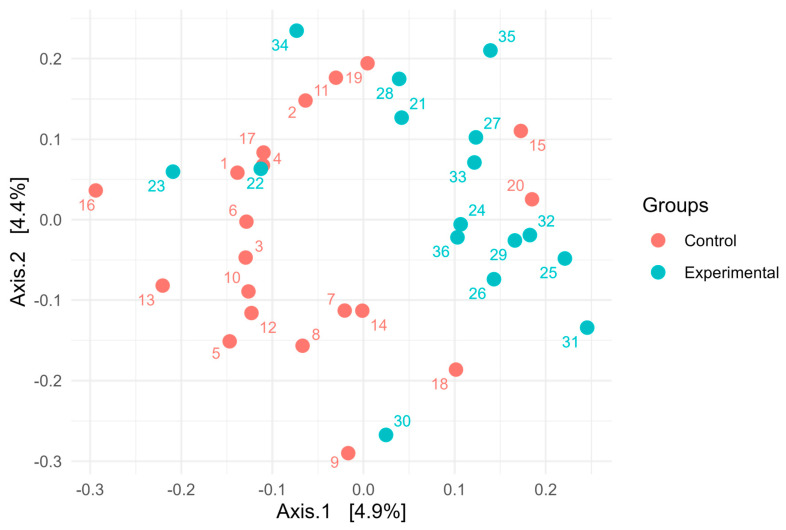
Principal Coordinates Analysis (PCoA) of Microbial Communities Based on Bray–Curtis Distance of cows fed a ration containing grass with and without combined inoculant.

16S rRNA gene sequencing of the rumen microbiota showed no significant differences in taxonomic composition between the control and experimental groups. At the phylum level (Figure 1), *Bacteroidota* was the dominant taxon across all samples, accounting for over 85% of the community, with slightly greater variability observed in the control group. *Firmicutes* was the second most abundant phylum, while other phyla such as *Fibrobacterota*, *Elusimicrobiota*, and *Patescibacteria* were present in relatively minor proportions. At the genus level (Figure 2), the microbial communities were dominated by members of the genus *Xylanibacter*. The next most abundant genera were *Prevotella*, *Succiciniclasticum,* and *Segatella*. The alpha diversity of microbial communities was assessed using observed richness (Figure 3A), the Simpson index (Figure 3B), the Shannon diversity index (Figure 3C), and the Fisher index (Figure 3D). Observed richness, reflecting the total number of ASVs detected, was similar between groups, with a median of approximately 415 in both control and experimental samples. The Simpson index (1 − D), which reflects the probability that two randomly selected individuals belong to different taxa, was high (median 0.9945), indicating high diversity. Shannon diversity, which combines both richness and evenness, showed comparable values across groups (median ~5.5), while the Fisher index, a richness estimator that accounts for the distribution of abundances, was around 70 in both groups. No statistically significant differences were observed between control and experimental samples for any of the indices (*p* > 0.05). Overall, these results indicate broadly similar alpha diversity patterns between the two groups, with high richness and evenness. Notably, although diversity metrics suggest a complex community structure, taxonomic bar plots revealed that approximately 70% of the community was assigned to a single genus, highlighting that high ASV-level diversity can agree with taxonomic dominance at higher ranks. Additionally, PERMANOVA using the Adonis test revealed statistically significant differences in microbial community composition between the control and experimental groups. Based on Jaccard distance (Figure 4), the group factor explained approximately 3.3% of the variance (R^2^ = 0.03328, F = 1.17, *p* < 0.01). Similarly, analysis using Bray–Curtis dissimilarity distance confirmed this finding (Figure 5), with group membership accounting for about 3.5% of the variation (R^2^ = 0.03466, F = 1.22, *p* < 0.01). However, despite reaching statistical significance, the low proportion of explained variance indicates that these differences are subtle and may have limited biological relevance. It is important to note that our results from 16S rRNA sequencing primarily detected changes in the resident microbial community and did not directly quantify the inoculated strains. The inoculant likely exerted its effect through metabolic reprogramming of rumen microbes (e.g., higher propionate production, reduced protozoa) rather than by establishing dominance in the rumen microbiota.

## 4. Discussion

The pH of silage is an essential factor influencing fermentation and the quality of ensiled fodder; a low pH is needed to preserve silage as it prevents its spoilage by inhibiting spoilage microorganisms [39]. While silage pH can be influenced by factors such as the type of forage and dry matter content, the presence of any silage additives plays a great role in pH regulation [40]. The reduced pH in this study can be linked to the presence of LAB in the inoculant [41], which also increased the lactic acid composition of the silage. Lactic acid (pH of 3.86), which LAB produces, is typically the acid present in silages at the highest concentration during ensiling and is 10–12 times stronger than any of the other major acids present in silages, such as acetic acid (pH of 4.75) and propionic acid (pH of 4.87) [40]. Our study confirms the claim that silage treated with LAB inoculants typically show both higher lactic acid concentrations and lower pH values after 30–60 days of ensiling, as the rapid accumulation of lactic acid overwhelms the system’s buffering capacity [42]. Furthermore, the pH drop caused by lactic acid accumulation inhibits protease activity and clostridial deamination, which could have contributed to the observed increase in the CP content of the silage [43]. By this inhibition, more true protein is preserved in the silage mass and less is converted to non-protein nitrogen (ammonia-N). Studies reported that LAB inoculation can significantly lower ammonia-N (AN/TN) and thereby maintain higher true-protein levels in alfalfa silages compared to untreated controls [44]. The insignificant effect of the combined inoculants on the silage FA profile, OM, DM, and NDF suggests that the inoculants’ action was highly targeted: it drove faster lactic-acid fermentation and protein preservation without altering the overall plant structural matrix or lipid fractions. In practical terms, this means that while nutrient preservation (protein) and fermentation quality improved, the bulk energy (OM, DM) and fiber composition remained constant, maintaining forage physical integrity and predictable fiber-driven rumen activity.

The comparison of the TMRs between the control and experimental groups after incorporating 230 g/kg of grass silage into the TMRs revealed only minor and non-significant differences in proximate composition, suggesting that the inclusion of the microbial inoculant during grass silage preparation did not alter the macronutrient content of the overall ration in a statistically meaningful way. Nevertheless, the numerical increase in crude protein content (+8 g/kg DM) in the experimental TMR could be indicative of improved forage fermentation quality, possibly due to the enhanced proteolysis inhibition with effective microbial inoculation [42]. Such shifts, although subtle, may contribute to improved digestibility and nitrogen utilization in the rumen.

Contrary to the reduced pH in the silage, ruminal pH increased, which can be linked to both the conversion of lactic acid to propionic acid in the rumen [40] and the presence of propionic bacteria in the inoculant. The observed increase in propionate reflects a redirection of fermentation end-products toward glucogenic pathways, which is consistent with reduced methane yield and improved energy efficiency. Furthermore, propionate production in the rumen uses protons (H^+^) rather than releasing them, unlike acetate and butyrate formation [45]. As a result, the overall acid load entering the rumen fluid is diminished, directly raising pH, resulting in increased ruminal propionate, and lowering the A:P ratio observed in this study. The reduction in valerate, branched-chain VFA (BCVFA), isovalerate, and ruminal NH_3_ strongly suggests reduced amino acid deamination in the rumen because VFAs like valerate, isovalerate, and other BCVFAs are produced when rumen microbes break down amino acids [46]. This observation reflects a more efficient capture of dietary N by microbes and less proteolysis. This result corroborates the finding of Jeong et al. [47] combining *L. plantarum* with propionate-producers (*Selenomonas ruminantium* and *Acidipropionibacterium thoenii*). It also complements the findings of Monteiro et al. [48] which reported that *L. plantarum* silage inoculation reduced ruminal NH_3_–N and BCVFA (including isovalerate) by limiting branched chain amino acid (BCAA) deamination and enhancing microbial nitrogen capture. Furthermore, because the ruminal protozoa population relies on ammonia and BCVFA for growth [49,50], the reduction in the total protozoa population reinforces the inhibition of BCAA deamination and proteolysis.

Studies have reported improvements in the lactation performance of cows fed silage inoculated with LAB [51] and propionic bacteria [52]. A review by Kung et al. [53] on studies using only *L. plantarum* as a silage inoculant reported an average increase of 1.2 kg of milk/d in the daily milk yield of cows consuming the inoculated silages, which aligns with the result of the current study. The observed increase in ruminal propionate (a primary gluconeogenic precursor) in this study provides more substrate for hepatic glucose synthesis, which results in not just milk yield but lactose synthesis in the mammary gland [54]. In addition, the improved CP composition of the silage provides a more efficient source of protein absorption in the small intestine, which supplies more absorbable amino acids to the mammary gland and results in higher milk CP and casein yields, as shown by the greater percentage of milk protein in this study.

Silage inoculant improves preservation of fermentable carbohydrate and protein, yielding substrates more accessible to rumen microbes [55]. This fosters greater cellulolytic and proteolytic bacterial growth, boosting total-tract digestibility of DM, OM, and CP. The observed reduction in the total protozoa count in the experimental group can also be connected to the improved digestibility and reduced CH_4_ production. Rumen protozoa harbor a large proportion of rumen methanogens and have a symbiotic relationship with them [56], with many methanogens adhering to or residing within protozoa, utilizing hydrogen produced by protozoal metabolism to generate methane [57,58]; thus, fewer protozoa often means fewer associated methanogens and less CH_4_ output [59]. This submission agrees with the report by Morgavi et al. [60] that studied the relationship between protozoa and methanogenesis. In addition to reducing protozoal populations, *Propionibacterium* strains in silage inoculants further aid CH_4_ mitigation by promoting propionate production, an alternative hydrogen sink that limits substrate availability for methanogenesis [27]. *P. acidipropionici* supplementation increased ruminal propionate and reduced methane output, reinforcing its role in lowering CH_4_ emissions through improved rumen fermentation pathways [26]. Adding to the shift in fermentation pathway, some studies have shown that a lower protozoa population is connected to improved nutrient digestibility [61,62]. Protozoa in the rumen consume bacteria, which are key contributors to microbial protein synthesis; thus, reducing protozoa can increase the flow of microbial protein to the small intestine, improving the host animal’s protein supply and nitrogen retention [63].

Beyond the overall reduction in protozoa, the decline in *Ophryoscolecidae*, the dominant family within the *Entodiniomorphid*, is particularly noteworthy. Members of this group are major contributors to hydrogen production and often serve as hosts for symbiotic methanogens [64,65]. Their suppression may therefore play a key role in reducing CH_4_ yield. In contrast, *Isotrichidae* preferentially ferment soluble sugars and store them as intracellular polysaccharides [66], favoring butyrate and lactate pathways and lowering A:P ratios [65,67]. Moreover, holotrichs are less frequently colonized by methanogens [64,68], and thus their increase may indicate a reduction in available ecological niches for methanogen proliferation. It is important to note, however, that the pattern observed in this study (higher ruminal pH, reduced *Ophryoscolecidae*, increased *Isotrichidae*, and decreased valerate) does not align with the findings of the meta-analysis by Dai et al. [69], which suggested a positive association with CH_4_ production. This discrepancy implies that additional mechanisms may underlie the observed reduction in CH_4_ emissions in our study.

The observed changes in milk fatty acid profile are likely due to the influence of the inoculants on silage fermentation and subsequent rumen microbial metabolism. As observed, LAB and propionic bacteria improved silage quality by increasing lactic and propionic acid production, which enhances silage preservation and alters the profile of fermentation end-products available to rumen microbes [6,70,71]. This shifted rumen fermentation toward higher propionate and lower acetate, influencing the biohydrogenation pathways of fatty acids. The increase in PUFAs and total UFA, including trans C18:1 isomers and n-3 fatty acids, suggests a partial inhibition of complete biohydrogenation, possibly due to changes in rumen microbial populations or activity [6,72]. The reduction in total SFA, especially C16:0, and the increase in C14:0 and C18:0, may reflect altered microbial conversion rates and desaturase enzyme activity in the mammary gland, as indicated by the increased desaturation index [6]. The lower n-6/n-3 ratio and increased n-3 content are consistent with improved silage quality and altered rumen fermentation, favoring the transfer of beneficial fatty acids into milk.

Supplementation with the compound microbial inoculant did not elicit significant shifts in the relative abundance of rumen microbiota. Nonetheless, four dominant bacterial genera, *Trueperella*, *Selenomonas*, *Succinivibrio*, and *Prevotella*, were consistently associated with fermentation pathways relevant to methane modulation. Despite taxonomic differences, these genera converge on a shared functional trait: the capacity to mitigate methane formation via enhanced propionate synthesis, thereby indirectly suppressing hydrogen availability for methanogenesis. Propionate is a key alternative electron sink during ruminal fermentation, and its biosynthesis consumes substantial NADH [73]. This metabolic route mitigates NADH overaccumulation and its oxidation, consequently limiting electron and proton release for hydrogenase-mediated H_2_ production, the primary substrate for methane synthesis [74]. The stoichiometric link between propionate formation and H_2_ utilization has been experimentally demonstrated: fermentation of one mole of glucose to propionate consumes one mole of hydrogen [75].

Independent studies corroborate the methane-mitigating potential of these genera. *Prevotella* and its associated enzymes were significantly enriched in low-emission ruminants, with the suppression of methane likely mediated through propionate production [76]. A meta-analysis revealed a strong negative association between *Succinivibrio* abundance and methane yield [77]. Mechanistically, *Succinivibrio* converts hydrogen to succinate, which undergoes decarboxylation to propionate [78], and its abundance correlates positively with propionate concentrations [79]. *Selenomonas* metabolizes lactate to propionate through sequential oxidation, carboxylation, reduction, dehydration, and decarboxylation steps [80], and its abundance is promoted by bromochloromethane or chloroform treatments that suppress methane formation by competing with methanogens for hydrogen [81,82]. Additionally, *Trueperella* abundance is inversely related to both raw and feed-intake-corrected methane yields in dairy cattle [83].

In the present trial, the pronounced reduction in methane emissions despite minimal taxonomic shifts suggests that the microbial additive acted primarily through metabolic reprogramming rather than community restructuring. Two non-exclusive pathways are proposed: (i) inoculated lactic acid bacteria competed for fermentable carbohydrates, producing lactate as a precursor for propionate biosynthesis [84], and (ii) inoculated propionate producers directly enhanced propionate output [85]. Together, these processes intensified hydrogen consumption, effectively constraining its availability for methanogenesis. Targeted enrichment of hydrogenotrophic microbes, such as propionate producers, is now recognized as a viable methane mitigation strategy [75], and the significant elevation of propionate concentrations in this study is fully consistent with such a mechanism.

A further notable outcome was the substantial decline in total protozoal abundance. As principal hydrogen producers within the rumen [60], protozoa generate H_2_ via anaerobic fermentation, subsequently supplying their endosymbiotic methanogens through interspecies hydrogen transfer [64]. While methanogen counts remained unchanged, the observed methane reduction is likely attributable to diminished hydrogen production and restricted methanogen colonization following protozoal suppression [86]. This hypothesis is consistent with prior evidence: protozoa-free sheep emit up to 43% less methane compared with controls [56], and meta-analytic synthesis confirms that protozoal depletion is generally accompanied by decreased methane emissions [87].

## 5. Limitations

This study has certain limitations that should be considered when interpreting the findings. First, only two silos (one control and one inoculated) were prepared, meaning that each treatment was represented by a single replicate at the silo level. As a result, differences in fermentation between silos could possibly be influenced by silo-specific factors aside from the inoculant. Second, only two ruminally cannulated cows per treatment were available for sample collection. The restricted number of cannulated animals was not due to careless experimental design but ethical limits in our study. While this may inevitably limit statistical power, we sought to maximize the robustness of the dataset by employing a repeated sampling strategy. Ruminal fluid was collected on four different days (days 1, 3, 6, and 8) and at three time points per day (pre-feeding at 6:00 h, and 3 and 6 h post-feeding). This repeated-measures approach increased the number of observations and provided a more reliable representation of rumen fermentation dynamics under the experimental conditions. Future studies with greater replication at both the silo and animal levels will be valuable to confirm and extend these findings.

## 6. Conclusions

Ensiling grass with a multi-strain inoculant comprising *Lactobacillus plantarum*, *L. buchneri*, *Propionibacterium acidipropionici*, and *P. thoeni* significantly improved silage fermentation quality, as indicated by the reduced pH and enhanced CP content. These improvements, which were incorporated at 230 g/kg of grass silage into the TMRs, translated into beneficial changes in rumen fermentation, which elevated the ruminal pH and propionate concentrations while reducing NH_3_ and protozoal abundance, thereby enhancing nutrient digestibility and lowering enteric CH_4_ emissions. In addition, cows fed the inoculated silage exhibited higher milk yield and energy-corrected milk output, along with improved milk composition characterized by higher milk protein content, elevated UFAs and CLA, and reduced SFAs. While 16S rRNA gene sequencing revealed only subtle shifts in microbial community structure, significant enrichment of propionate-associated genera supports a metabolic basis for the observed reductions in methane. Collectively, these findings revealed the inoculant’s potential as a multifunctional strategy to improve silage preservation, optimize rumen function, enhance milk quality, and reduce environmental impact, supporting its application in sustainable dairy production systems.

## Figures and Tables

**Table 1:** Chemical composition, fermentation characteristics, and fatty acid profile of grass silage with and without combined inoculant (*n* = 3 samples per treatment).

Item	Control	Experimental	SEM	*p* Value
pH	5.06	4.56	0.13	0.02
Dry matter (DM) (g/kg)	436	413	10.6	0.34
Organic matter (g/kg DM)	881	873	4.85	0.46
Crude protein (g/kg DM)	111	129	4.71	0.05
Neutral detergent fiber (g/kg DM)	524	517	12.1	0.72
Crude fat (g/kg DM)	25.3	26.1	0.61	0.55
Crude ash (g/kg DM)	86.1	100	4.03	0.46
Fermentation characteristics (g/kg DM)				
Lactic acid	56.8	78.3	5.57	0.03
Acetic acid	25.6	29.0	4.58	0.76
Propionic acid	4.61	7.62	0.70	0.01
Butyric acid	1.22	0.85	0.10	0.06
Fatty acid composition (g/100 g total FA)				
C12:0	0.16	0.27	0.11	0.52
C14:0	0.52	0.60	0.19	0.36
C16:0	21.1	20.5	1.89	0.81
C16:1	1.82	1.76	0.36	0.22
C18:0	4.02	4.33	0.62	0.37
C18:1cis 9	3.99	4.44	0.23	0.11
C18:2 cis 9 cis 12	13.9	14.4	0.92	0.64
C18:3 cis 9 cis 12 cis 15	46.2	45.5	1.12	1.27
Others *	8.29	8.20	0.83	0.83

SEM-standard error of mean; Others *-other fatty acids: C14:1, C15:1. C17:0, C17:1, C18:1 trans-6-8, C18:1 trans-9, C18:1 cis-11, C18:1 cis-12, C18:1 cis-13, C18:1 cis-14, C20:0, C20:1 trans, C18:3 n-6, C21:0, C20:2, C22:0, C20:3 n-6, C22:1 n-9, C20:3 n-3, C23:0, C22:2, C24:0, C24:1, and C22:5n3.

**Table 2:** Chemical composition and fatty acid profile of total mixed rations (TMRs) with and without combined inoculant (*n* = 3 samples per treatment).

Item	Control	Experimental	SEM	*p* Value
Dry matter (DM) (g/kg)	438	427	9.79	0.74
Organic matter (g/kg DM)	962	964	0.62	0.15
Crude protein (g/kg DM)	156	164	2.15	0.17
Neutral detergent fiber (g/kg DM)	353	348	8.79	0.19
Fatty acid composition (g/100 g total FA)				
C12:0	0.41	0.23	0.06	0.15
C14:0	0.82	0.77	0.02	0.35
C16:0	18.30	18.84	0.21	0.23
C16:1	0.86	0.88	0.01	0.54
C18:0	3.05	3.07	0.06	0.84
C18:1cis 9	19.41	19.23	0.28	0.78
C18:2 cis 9 cis 12	39.49	39.02	0.34	0.53
C18:3 cis 9 cis 12 cis 15	13.40	13.65	0.71	0.87
Others *	4.27	4.30	0.03	0.68

SEM-standard error of mean; Others *-other fatty acids: C10:0, C14:1, C15:0, C15:1, C17:0, C17:1, C20:0, C18:3 n-6, C21:0, C20:2, C22:0, C20:3 n-6, C22:1 n-9, C20:3 n-3, C20:4 n-6, C23:0, C22:2, C24:0, C20:5 n-3, C24:1, C22:5n3 and C22:6 n-3.

**Table 3:** Basic rumen parameters of cows fed grass silage with and without combined inoculant (*n* = 2 cannulated cows per treatment; 4 days; 3 daily subsamples).

Item	Control	Experimental	SEM	*p* Value
pH	5.83	6.06	0.04	0.03
Acetate (A) (mM)	75.4	73.5	5.39	0.17
Propionate (P) (mM)	26.3	28.3	2.86	0.03
Isobutyrate (mM)	1.30	1.33	0.11	0.42
Butyrate (mM)	18.9	19.2	2.37	0.77
Isovalerate (mM)	2.02	1.57	0.67	0.02
Valerate (mM)	1.82	1.41	0.23	0.01
Total VFA (mM)	125	125	9.66	0.75
A:P Ratio	2.87	2.60	0.26	0.01
NH_3_ (mM)	8.67	7.61	0.22	0.02
Isotrichidae (×10^3^/mL)	9.98	12.8	0.32	0.03
Ophryoscolecidae (×10^5^/mL)	1.56	1.08	0.05	0.03
Total Protozoa (×10^5^/mL)	1.66	1.21	0.05	0.03

**Table 4:** Effect of grass silage with and without combined inoculant on lactation performance (*n* = 12 cows per treatment; 4 days; 2 daily subsamples).

Item	Control	Experimental	SEM	*p* Value
Dry matter intake (kg/day)	24.0	24.5	0.10	0.19
Milk yield (kg/day)	33.5	35.3	0.44	0.04
ECM (kg/day)	35.3	37.2	0.52	0.05
Basal milk composition (%)				
Dry matter	13.5	13.6	0.15	0.79
Crude fat	4.32	4.18	0.10	0.45
Crude protein	3.64	3.87	0.05	0.01
Caseins	2.94	3.04	0.04	0.26
Lactose	4.64	4.76	0.02	0.01
Urea (mg/L)	203	223	5.11	0.04
Daily milk solids (kg/day)				
Crude fat	1.44	1.47	0.03	0.58
Crude protein	1.22	1.36	0.03	0.01
Caseins	0.98	1.07	0.02	0.01
Lactose	1.56	1.68	0.02	0.01

SEM-standard error of mean; ECM: Energy-corrected milk calculated according to the following equation: ECM = milk yield (kg) × (38.3 × fat (g/kg) + 24.2 × protein (g/kg) + 783.2)/3140 [38].

**Table 5:** The effect of grass silage ensiled with and without combined inoculant on methane (CH_4_) production (*n* = 2 cannulated cows per treatment; 4 days; 3 daily subsamples) and digestibility (*n* = 2 cannulated cows per treatment; 8 days).

Item	Control	Experimental	SEM	*p* Value
Dry matter intake (DMI)	24.0	24.5	0.10	0.19
Total-tract digestibility, g/kg DM				
Dry matter	629	650	5.39	0.04
Organic matter	690	712	5.33	0.04
Crude protein	596	636	6.95	0.01
CH_4_ g/d	397	368	3.31	0.01
CH_4_ g/kg DMI	16.5	15.1	0.18	0.01

**Table 6:** Fatty acid profile in milk from cows fed a ration containing grass with and without combined inoculant (*n* = 12 cows per treatment; 4 days; 2 daily subsamples).

Fatty Acids Specified (g/100 g Fatty Acid Methyl Esters (FAME)	Control	Experimental	SEM	*p* Value
Saturated fatty acids (SFA)				
C12:0	4.69	5.04	0.20	0.22
C14:0	14.1	14.6	0.28	0.02
C15:0	1.37	1.50	0.06	0.66
C16:0	36.7	32.9	0.79	0.01
C18:0	9.37	10.1	0.26	0.03
Monounsaturated fatty acids (MUFA)				
C16:1	1.83	1.76	0.06	0.50
C18:1 trans-10	0.51	0.56	0.02	0.02
C18:1 trans-11	0.79	1.03	0.03	0.01
C18:1 cis-9	20.6	21.4	0.37	0.06
Polyunsaturated fatty acids (PUFA)				
C18:2 cis-9 cis-12	2.68	2.60	0.09	0.02
C18:2 cis-9 trans-11	0.48	0.60	0.02	0.01
C18:2 trans-10 cis-12	0.14	0.15	0.00	0.01
C18:3 cis-9 cis-12 cis-15	0.49	0.40	0.01	0.01
C20:4 n-6	0.03	0.04	0.01	0.07
C20:5 n-3	0.04	0.06	0.01	0.04
C22:6 n-3	0.01	0.09	0.05	0.84
Others ^1^	6.22	7.16	0.16	0.11
Total SFA	67.2	65.2	0.52	0.02
Total UFA	32.8	34.8	0.52	0.02
Total MUFA	28.5	30.3	0.44	0.01
Total PUFA	4.35	4.49	0.17	0.13
Total trans-C18:1	1.96	2.33	0.05	0.01
Total MCFA	60.5	57.6	0.71	0.01
Total LCFA	39.5	42.4	0.71	0.01
n-6	6.04	5.95	0.18	0.03
n-3	0.68	0.71	0.10	0.01
PUFA/SFA	0.06	0.07	0.00	0.65
n-6/n-3	8.97	10.1	0.28	0.01
Desaturation indexes (DI)				
DI	0.29	0.30	0.00	0.02
DI C14:1	0.11	0.09	0.00	0.01
DI C16:1	0.05	0.07	0.01	0.13
DI C18:1	0.69	0.68	0.01	0.23
Thrombogenic index	3.36	3.13	0.08	0.10
Atherogenic index	3.01	2.82	0.07	0.11

^1^ Other fatty acids: C14:1. C15:1. C17:0. C17:1. C18:1 trans-6-8. C18:1 trans-9. C18:1 cis-11. C18:1 cis-12. C18:1 cis-13. C18:1 cis-14. C20:0. C20:1 trans. C18:3 n-6. C21:0. C20:2. C22:0. C20:3 n-6. C22:1 n-9. C20:3 n-3. C23:0. C22:2. C24:0. C24:1, and C22:5n3.

## Data Availability

The 16S rRNA sequencing data generated in this study have been deposited in the NCBI Sequence Read Archive (SRA) under BioProject ID PRJNA1308859. Accession numbers for individual sequencing runs (FASTQ files) are available through this BioProject.

## Abbreviations

The following abbreviations are used in this manuscript:

LAB	Lactic acid bacteria
FA	Fatty acid
UFA	Unsaturated fatty acid
SFA	Saturated fatty acid
PUFA	Polyunsaturated fatty acid
MUFA	Monounsaturated fatty acid
VFA	Volatile atty acid
CH_4_	Methane
DMI	Dry matter intake
DM	Dry matter
OM	Organic matter
CP	Crude protein
RF	Rumen fluid
H_2_	Hydrogen
